# A Survey of Avian Influenza in Tree Sparrows in China in 2011

**DOI:** 10.1371/journal.pone.0033092

**Published:** 2012-04-04

**Authors:** Yan Han, Guangyu Hou, Wenming Jiang, Chunhua Han, Shuo Liu, Jie Chen, Jinping Li, Peng Zhang, Baoxu Huang, Yuehuan Liu, Jiming Chen

**Affiliations:** 1 China Animal Health and Epidemiology Center, Qingdao, China; 2 College of Animal Science and Veterinary Medicine, Qingdao Agricultural University, Qingdao, China; 3 Institute of Animal and Husbandry Medicine, Beijing Academy of Agriculture and Forestry Sciences, Beijing, China; Centers for Disease Control and Prevention, United States of America

## Abstract

Tree sparrows (*Passer montanus*) are widely distributed in all seasons in many countries. In this study, a survey and relevant experiments on avian influenza (AI) in tree sparrows were conducted. The results suggested that the receptor for avian influenza viruses (AIVs), SAα2,3Gal, is abundant in the respiratory tract of tree sparrows, and most of the tree sparrows infected experimentally with two H5 subtype highly pathogenic avian influenza (HPAI) viruses died within five days after inoculation. Furthermore, no AIVs were isolated from the rectum eluate of 1300 tree sparrows, but 94 serological positives of AI were found in 800 tree sparrows. The serological positives were more prevalent for H5 subtype HPAI (94/800) than for H7 subtype AI (0/800), more prevalent for clade 2.3.2.1 H5 subtype HPAI (89/800) than for clade 2.3.4 (1/800) and clade 7.2 (4/800) H5 subtype HPAI, more prevalent for clade 2.3.2.1 H5 subtype HPAI in a city in southern China (82/800) than in a city in northern China (8/800). The serological data are all consistent with the distribution of the subtypes or clades of AI in poultry in China. Previously, sparrows or other passerine birds were often found to be pathogenically negative for AIVs, except when an AIV was circulating in the local poultry, or the tested passerine birds were from a region near waterfowl-rich bodies of water. Taken together, the data suggest that tree sparrows are susceptible to infection of AIVs, and surveys targeting sparrows can provide good serological data about the circulation of AIVs in relevant regions.

## Introduction

Tree sparrows (*Passer montanus*) in the family *Passeridae* and the order *Passeriformes* are small, plump, brown-grey passerine birds with short tails and stubby beaks. They are widely distributed in China and many other countries in the world [1]. Tree sparrows are primarily seed-eaters, although they also consume small insects. Many tree sparrows scavenge for food around farms, villages and cities [1]. Tree sparrows are sold in pet bird markets in many cities in China, mainly for bird releasing of Buddhists.

Avian influenza viruses (AIVs), which have caused huge economic losses to the global poultry industry and posed a great threat to global public health in recent years [2], [3], have been isolated in tree sparrows and related passerine birds [4]–[11]. Tree sparrows, if they are susceptible to infection of AIVs, should be of significance to be targeted for surveys, to provide epidemiological data on circulation of AIVs in relevant regions, because they are abundant, widespread, unvaccinated, and come into frequent contact with the local poultry. They are also easily available in pet bird markets in China. However, despite considerable effort for study of AIVs in wild birds in many countries [12]–[15], systematic surveys of AIVs in tree sparrows are still scarce.

In this report, we conducted a survey and relevant experiments about avian influenza (AI) in tree sparrows in China. We firstly determined the type of influenza virus receptor in the respiratory tract of tree sparrows using an immunohistochemical staining method. Then, we examined whether tree sparrows are susceptible to challenge of H5 highly pathogenic avian influenza (HPAI) viruses. This was followed by a survey of AI in tree sparrows with detection of 1300 rectum eluate samples and 800 serum samples collected from tree sparrows in two cities in China.

## Materials and Methods

### Ethics statement

This study was conducted according to the guidelines of animal welfare of World Organization for Animal Health [16], and approved by the Animal Welfare Committee of China Animal Health and Epidemiology Center (Permit number: 2011-CAHECAW-02).

### Animals

A total of 1360 tree sparrows were bought for this study in the April, 2011. Among them, 860 tree sparrows were bought from Nanshan Pet Market in Qingdao, a city in northern China. Two of these birds were used for the receptor study, 28 for red blood cell (RBC) collection, 30 for the virus challenge experiment, 400 for collection of rectum eluate samples, and the remaining 400, which were apparently healthy, were used for collection of both rectum eluate and serum samples. Another 500 were bought from Huadiwan Pet Market in Guangzhou, a city in southern China. Among them, 100 were used for collection of rectum eluate samples, and the remaining 400, which were apparently healthy, were used for collection of both rectum eluate and serum samples. The tree sparrows were originally caught from the countryside near the cities of Qingdao and Guangzhou, respectively.

### Receptor study

Sialic acid linked to galatose by an α-2,3 linkage (SAα2,3Gal), the receptor for AIVs, and sialic acid linked to galatose by an α-2,6 linkage (SAα2,6Gal), the receptor for human influenza viruses, on the surface of the trachea and larynx of tree sparrows, were detected by immunohistochemical staining. The method utilizes two kinds of digoxigenin-labeled lectins which can bind specifically to SAα2,3Gal and SAα2,6Gal. Firstly, the larynx and trachea (including upper, middle, and lower parts) were collected from two apparently healthy tree sparrows which were euthanatized by intravenous administration of sodium pentobarbital (100 mg/kg body weight). The tissues were fixed with 10% neutral formalin for 24 h. They were then trimmed and fixed for an additional 12 h in fresh 10% neutral formalin. This was followed by dehydration and paraffin embedding. The embedded tissues were cut into 3 mm sections using a microtome. The mounted sections were deparaffinized in xylene and immersed in water. Then, the sections were detected using the DIG Glycan Differentiation Kit (Roche Applied Science) according to the kit protocol. Briefly, three pairs of the sections were incubated with 1∶50 dilution of digoxigenin-labeled *Maackia amurensis* lectin, 1∶50 dilution of digoxigenin-labeled *Sambucus nigra* lectin, or Tris-buffered saline (TBS, pH 7.6), at 35°C for 1 h. After washing three times with TBS (pH 7.6), the sections were incubated with anti-digoxigenin (anti-DIG) antibodies that are conjugated to alkaline phosphatase at 35°C for 30 min. This was followed by washing three times with TBS (pH 7.6) and immunohistochemical staining using nitro-blue tetrazolium chloride (NBT) and 5-bromo-4-chloro-3′-indolyphosphate p-toluidine salt (BCIP). The sections stained with black-purple, black-blue, pure blue precipitate were judged as strong positives, positives and negatives, respectively.

### Virus propagation

Two H5 subtype HPAI viruses, A/chicken/Hubei/QE8/2009(H5N1) and A/chicken/Shanxi/QX2/2009(H5N1), were isolated during a survey of HPAI in poultry in China in 2009 [17]. They belong to clades 2.3.2.1 and 7.2, respectively, according to an updated nomenclature system published in 2012 [18]. Both of them are lethal for ten of ten 4-week-old Specific Pathogen Free (SPF) chickens within 10 days following intravenous inoculation with 0.2 mL of a 1/10 dilution of a bacteria-free, infective allantoic fluid. The stocks of the two viruses were produced by passage in 10 day-old SPF embryonated chicken eggs, and titrated by passage in 10 day-old SPF embryonated chicken eggs after 10-fold serial dilution (ten embryonated eggs for each dilution). Allantoic fluid from inoculated eggs was diluted in sterile phosphate-buffered solution (PBS, pH 7.2) to obtain a final inoculum titer of 10^5.0^ median embryo lethal dose (ELD_50_) per bird.

### Animal challenge experiment

Thirty apparently healthy adult tree sparrows were randomly divided into three groups, each including ten sparrows. Group 1 and group 2 were inoculated with two H5 subtype HPAI viruses, A/chicken/Hubei/QE8/2009(H5N1) and A/chicken/Shanxi/QX2/2009(H5N1), respectively. Each bird was inoculated intranasally with 10^5.0^ ELD_50_ virus. Group 3 was set as the blank control and each bird was inoculated intranasally with a sham inoculum which was made using sterile allantoic fluid diluted 1∶100 in sterile PBS (pH 7.2). The animal challenge test was done in an Animal Biosafety Level 3 (ABSL-3) laboratory. The birds were examined every 12 h after virus inoculation, and dead birds were stored at −70°C until collection of rectum eluate and organs for testing. Five days after virus inoculation, the remaining live birds were euthanatized by intravenous administration of sodium pentobarbital (100 mg/kg body weight). The rectum eluate samples from all the birds were collected and tested using the methods given below. The trachea, lung, liver, spleen, and rectum were also collected from the birds, and the tissues from the same bird were pooled and homogenized in a 10-fold volume of PBS (pH 7.2) containing 5000 units/mL of penicillin and 5 mg/mL of streptomycin. The homogenates were clarified by centrifugation at 8000 g for 10 min, and the supernatants were inoculated into 10-day-old SPF embryonated eggs. After a 3-day incubation at 37°C, the allantoic fluid was tested using the method given below.

### Rectum eluate sample collection and detection

Because common swabs are too large for tree sparrows, rectum eluate samples were collected instead for virus detection. Briefly, 150 µL PBS (pH 7.2) was injected gently into the rectum through the bird cloaca using a 200 µL tip and a 200 µL pipette. Ten seconds later, no less than 80 µL fluid was withdrawn from the cloaca with gentle suction using the same tip and pipette, and the fluid was diluted in 1 mL PBS added with streptomycin (500 mg/L, final) and gentamycin (250 mg/L, final), and used as the rectum eluate sample. The tree sparrows, if only subject to rectum eluate sample collection, were released back to nature thereafter.

The rectum eluate samples were clarified by centrifugation at 8000 g for 10 min,, and the supernatants were inoculated in SPF chicken embryonated eggs via the allantoic sac route. The eggs were further incubated for four days at 35°C. Thereafter, the allantoic fluids of the embryos were tested using the hemagglutination assay using chicken RBCs. All the hemagglutination-positive samples and the allantoic fluids of the embryos which died during the incubation, were investigated further using the universal RT-PCR, which targets the whole length of the HA gene of all subtypes of influenza viruses [19]. RT-PCR was performed in a 50 µL reaction mixture containing 5 µL template RNA, 25 µL One-step RT-PCR buffer, 1 µL of each primer (20 µM) and 2 µL One-step RT-PCR enzyme mix. The mixture was initially incubated at 50°C for 30 min and denatured at 94°C for 2 min, and then 30 cycles were performed with denaturation at 94°C for 30 s, annealing at 55°C for 30 s and extension at 72°C for 2 min. RT-PCR products were examined using agar gel electrophoresis and automatic sequencing.

### Standard antigen preparation

Four recombinant viruses, H7-7.2.3, H5-2.3.4, H5-2.3.2.1 and H5-7.2 were prepared from synthesized DNA sequences using the reverse genetics technology according to the method described previously [20]. They share the same internal genes of the PR8 influenza virus strain (GenBank accession numbers: NC00201, NC002019–NC002023). Their hemagglutinin (HA) and neuraminidase (NA) gene sequences were synthesized according to the ones of A/chicken/Hebei/1/2002(H7N2) (GenBank accession numbers: AY724257 and AY724264), A/duck/Liaoning/Q1/2009(H5N1) (GenBank accession numbers: HM006795 and JN790583), A/chicken/Hubei/QE8/2009(H5N1) (GenBank accession numbers: HM006817 and JN790582), A/chicken/Shanxi/QX2/2009(H5N1) (GenBank accession numbers: HM583608 and JN790584), except that only two alkaline amino acid residues were kept at the cleavage site of the HA genes to minimize the pathogenicity of the recombinant viruses. The recombinant viruses were propagated in 10 day-old SPF embryonated chicken eggs. Allantoic fluid from inoculated eggs was inactivated using formaldehyde (0.2% v/v, final). The inactivated allantoic fluid was used as the antigen for detection of antibodies against relevant clades of AIVs, after standardization using standard positive serum samples bought from Harbin Weike Biotechnology (Harbin, China) and a batch of 100 standard negative serum samples prepared from SPF chicken in house. Antigen H7-7.2.3 was used for detection of specific antibodies against clade H7.2.3 of H7 subtype AIVs which is the dominant lineage of H7 subtype AIVs in the Eastern Hemisphere in recent years [21]. Antigens H5-2.3.4, H5-2.3.2.1, H5-7.2 were used for detection of specific antibodies against clades 2.3.4, 2.3.2.1 and 7.2 of H5 subtype AIVs, respectively.

### Serum sample collection and detection

Serum samples from tree sparrows were collected from the jugular vein using 1 mL syringes. We found that collecting serum samples from the jugular vein is much easier and less harmful to tree sparrows than from the wing vein. Serum samples were prepared by separating the sera from the blood after clotting. The titer of the serum antibodies against AIVs were examined using the hemagglutination inhibition (HI) assay. Firstly, 25 µL of PBS was dispensed into each well of a plastic V-bottomed microtiter plate, and 25 µL of serum was placed into the first well of the plate, and then 2-fold diluted serially across the plate. This was followed by adding four hemagglutinating unit (HAU) of the standard antigen in 25 µL to each well and incubation for 30 min at room temperature (about 20°C). Then, 25 µL of 1% (v/v) tree sparrow RBCs was added to each well. After gentle mixing, the RBCs were allowed to settle for 30 min at room temperature. The blank control (without sera), the standard positive and negative controls (using chicken positive and negative sera) sera were set for the HI assay. Serological positives were the ones with a HI titer ≥16, and if a serum sample was of a HI titer ≥16 to two or three clades of H5 subtype HPAI viruses, the sample was considered as the positive of the clade to which the sample was of the highest HI titer, rather than the positive of the other clades.

## Results

### Receptor on the surface of the trachea and larynx of tree sparrows

**Figure 1:**
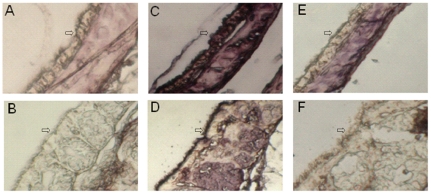
Immunohistochemical staining for detection of influenza virus receptor on the surface of the trachea and larynx of tree sparrows. Pair A & B: the negative controls not treated with any lectin; Pair C & D: treated with *Maackia amurensis* lectin which can bind to SAα2,3Gal; Pair E & F: treated with *Sambucus nigra* lectin which can bind to SAα2,6Gal; A, C, E: the trachea tissue sections; B, D, F: the larynx tissue sections. The tissue parts (shown with arrows) in Pair C & D rather than their counterparts in Pair A & B and Pair E & F were stained black-purple, indicating that the receptor for AIVs, SAα2,3Gal, is abundant on the surface of the trachea and larynx of tree sparrows.

As shown in Fig. 1, the sections of the trachea and larynx of tree sparrows treated with *Maackia amurensis* lectin were stained black-purple (strong positive), while their counterparts treated with *Sambucus nigra* lectin were stained similar to the negative controls which were not treated with any lectin. Because *Maackia amurensis* lectin and *Sambucus nigra* lectin can bind specifically to SAα2,3Gal and SAα2,6Gal, respectively, the results indicate that the receptor for AIVs, SAα2,3Gal, rather than the receptor for human influenza viruses, SAα2,6Gal, is abundant on the surface of the trachea and larynx of tree sparrows.

### Survival of the challenged animals

Ten, six and two tree sparrows in Groups 1, 2 and 3 died within 5 days after inoculation, respectively. Clade 2.3.2.1 virus was isolated from the tissue and rectum eluate samples of all the ten dead sparrows in Group 1, and clade 7.2 virus was isolated from the tissue and rectum eluate samples of four of the six dead sparrows in Group 2. No AIVs were isolated from any samples of the remaining birds in Group 2 and any birds in Group 3. All the isolated viruses were confirmed through sequencing of the viral HA gene.

### Detection of survey samples

There were no AIVs isolated from the rectum eluate of the 1300 tree sparrows in the serological survey, even though the allantoic fluid was subject to secondary passage in SPF embryonated chicken eggs. In contrast, as shown in Table 1 and Table S1, 11.75% of the serum samples tested positive by the HI assay. The serological positives were more prevalent for H5 subtype HPAI (94/800) than for subtype H7 HPAI (0/800), more prevalent for clade 2.3.2.1 H5 subtype HPAI (89/800) than for clade 2.3.4 (1/800) and clade 7.2 (4/800), and more prevalent for clade 2.3.2.1 H5 subtype HPAI in the city of Guangzhou in southern China (81/800) than in the city of Qingdao in northern China (8/800). The HI titers of the serological positives were moderately elevated (never exceeding 64), and only four serum samples reacted to more than one clade with a HI titer ≥16 (Table S1).

**Table 1:** Numbers of serological positives of different subtypes or clades of avian influenza in tree sparrows bought from two cities.

Detection antigen	Cities	
	Qingdao (n = 400)	Guangzhou (n = 400)
H5-2.3.2.1	8	81
H5-2.3.4	0	1
H5-7.2	2	2
H7-7.2.3	0	0

In addition, all the positive and negative controls of chicken sera used in the HI assay were detected using sparrow RBCs as positives and negatives, respectively. They were of the same HI titers as detected using chicken RBCs. Furthermore, we randomly selected 10 positive tree sparrow sera and 20 negative tree sparrow sera identified in this study using the H5-2.3.2.1 antigen and sparrow RBCs, and re-tested them using the same antigen and chicken RBCs. We observed only minor variation in HI titers (24 equivalent and 6 with one dilution difference).

## Discussion

Many avian species, such as chickens, ducks and geese, contain predominantly SAα2,3Gal, the receptor for AIVs, in the upper respiratory tract or in the intestines, and so they are susceptible to AIV infection [22]. In contrast, humans are relatively insusceptible to AIV infection because they have primarily SAα2,6Gal, the receptor for human influenza viruses, in the upper respiratory tract. Pigs are unusual in that they have abundant SAa2,3Gal and SAa2,6Gal in their respiratory tracts, suggesting that pigs can serve as a mixing vessel for generating human influenza virus and AIV re-assortment strains, which could result in pandemic outbreaks of influenza virus in humans [23]. Pigeons are also unusual because they have abundant SAα2,6Gal and little SAα2,3Gal on the epithelial surfaces of the larynx, trachea, bronchus, and bronchiole, which can explain why pigeons are naturally resistant to AIV infection [22]. As far as we know, this is the first report showing that SAα2,3Gal is abundant in the respiratory tract of tree sparrows, indicating that tree sparrows are likely susceptible to AIV infection.

The animal challenge experiment further suggest that tree sparrows are likely susceptible to AIV infection, because more tree sparrows inoculated with H5 subtype HPAI viruses died than the ones inoculated with the sham inoculum, and the viruses were isolated from most (14/20) of the tree sparrows inoculated with the H5 subtype HPAI viruses. Some previous reports also supported that tree sparrows and related passerine birds are susceptible to infection of AIVs through surveys or animal experiments [4]–[11], [24]–[29]. Some previous studies found that sparrows infected with AIVs did not support close-contact transmission [5], [26], [29], [30], suggesting that they shed few viruses in the environment after infection, and thus they are more likely to be the victim than the vector in the transmission of the viruses.

A few tree sparrows died within five days after inoculation with a sham inoculum in the animal challenge experiment. This might result from that they were under great stress in the experimental environment. Additionally, the animal challenge experiment demonstrated that AIVs could be isolated from the rectum eluate of infected sparrows, which supports the virus detection method used in this study.

Natural infection of AIVs in sparrows or related passerine birds has been identified in the past for multiple times [4]–[11]. For example, in 1985, serological evidence of H7N7 HPAI virus infection in sparrows was found in a region of Australia with H7N7 HPAI outbreaks in the local poultry [5]. In 2004, four H5 subtype HPAI viruses were isolated from 38 tree sparrows in China, and in that year H5 subtype HPAI outbreaks frequently occurred in China [7]. As tree sparrows are susceptible to AIVs, and they are abundant, widespread, and free ranging, it is possible for them to be infected with AIVs, if an AIV is circulating in poultry or other wild birds in the region where they live. On the other side, some surveys published previously plus the one reported here also suggested that it was uncommon to isolate AIVs from sparrows or other birds in *Passeridae* or *Passeriformes* [31]–[41]. For example, in 1999–2000, no AIVs were isolated from passerine birds through a large-scale surveillance of AIVs in wild birds conducted in North Europe [33]. In 2004–2007, a large-scale survey conducted in China identified 149 AIV positive samples in six orders of wild birds, with the lowest prevalence of AIVs in *Passeriformes* (0.36%) [35]. These negative data may partially be explained by the fact that sparrows and other birds in *Passeridae* or *Passeriformes* are usually terrestrial, and thus have fewer chances to be infected through contaminated water compared to waterfowls. In general, the data published previously and reported here support the notion that it is uncommon to isolate AIVs from sparrows or related passerine birds, except when an AIV is circulating in the local poultry [5]–[7], [9], or the tested passerine birds are from a region near waterfowl-rich bodies of water [8], [28].

The serological data reported here are consistent with the distribution of H5 and H7 AIVs in poultry in China in recent years, and this, in turn, supports the serological methods used in this study. [17]. Firstly, as far as we know, to date, only one flock infected with H7 subtype low pathogenic AIV has been identified through massive surveillance programs conducted in 1995–2011 in China [42], which is supported by the absence of H7 serological positives in this survey. Secondly, it has been well recognized that the risk of H5 subtype HPAI in southern China is higher than in northern China, because ducks, open water bodies and live bird markets are all of a higher density in southern China than in northern China [43]. This is also in agreement with our findings that H5 subtype HPAI serological positives were more prevalent in the tree sparrows collected in southern China than in the tree sparrows collected in northern China. Thirdly, three clades of H5 subtype AIVs, i.e. clades 2.3.4, 2.3.2.1 and 7.2, co-circulated in China in recent years, and clade 2.3.2.1 had become the most dominant lineage by 2009 [17]. This is consistent with that much more serological positives were found for clade 2.3.2.1 than for the other two clades (Table 1). clade 2.3.2.1 has been detected in nine countries since 2007 (China, Japan, South Korea, Russia, Mongolia, Vietnam, Bhutan, Israel and Romania), and it is the second clade of H5 subtype HPAI viruses having spread from Asia to Europe in recent years [17], [18]. The dominance and wide spreading of clade 2.3.2.1 indicate that this clade is likely of more fitness than other clades.

The majority of positive sera titers identified in this study are not high, and one possible explanation for this could be that the birds were only infected with a small number of infectious H5 subtype HPAI virus particles. This may also partially explain why the birds were able to survive infection with the deadly viruses. In addition, not all the tree sparrows infected with the highly pathogenic viruses died in the animal experiment reported here, and this may be also true in the field as suggested by the results of our serological survey.

We used sparrow RBCs rather than chicken RBCs in the serological survey, because it was stated in a protocol issued by China Ministry of Agriculture in 2007, that homogenous RBCs may be better than heterogeneous RBCs, in minimizing false positives. This notion was also supported by an academic meeting report in China in 2005 (unpublished).

Based on data from receptor distribution staining and animal challenge experiments, this study suggests that tree sparrows are susceptible to infection of AIVs. Additionally, surveys targeting tree sparrows can provide good serological data about the circulation of AIVs in relevant regions, although it is uncommon to isolate AIVs from apparently healthy tree sparrows.

## Supporting Information

Table S1 **The titers of the positive sera against three clades of H5 subtype avian influenza viruses.**
(DOC)
