# Supplementary material for: A Survey of Avian Influenza in Tree Sparrows in China in 2011
Source: PLoS One. 2012 Apr 4;7(4):e33092. doi: 10.1371/journal.pone.0033092 (PMC3319536; doi:10.1371/journal.pone.0033092)
Supplement: Table S1 — The titers of the positive sera against three clades of H5 subtype avian influenza viruses. (DOC) [file pone.0033092.s001.doc]

Table S1

The titers of the positive sera against three clades of H5 subtype avian influenza viruses (the ones judged as positives to Clades 2.3.2, 2.3.4 and 7 are shown in light yellow, light blue and pink shallow, respectively).

| Sera Number | Antigen Used | | |
| --- | --- | --- | --- |
|  | H5-2.3.2 | H5-2.3.4 | H5-7 |
| Guangzhou 9 | 64 | 2 | 2 |
| Guangzhou 11 | 16 | 2 | 0 |
| Guangzhou 15 | 16 | 2 | 0 |
| Guangzhou 16 | 32 | 0 | 0 |
| Guangzhou 18 | 16 | 0 | 0 |
| Guangzhou 23 | 32 | 0 | 2 |
| Guangzhou 24 | 32 | 0 | 2 |
| Guangzhou 25 | 16 | 0 | 0 |
| Guangzhou 43 | 16 | 0 | 0 |
| Guangzhou 46 | 64 | 2 | 0 |
| Guangzhou 48 | 16 | 0 | 0 |
| Guangzhou 61 | 64 | 2 | 0 |
| Guangzhou 62 | 32 | 0 | 0 |
| Guangzhou 69 | 16 | 0 | 0 |
| Guangzhou 78 | 32 | 4 | 2 |
| Guangzhou 79 | 16 | 2 | 2 |
| Guangzhou 85 | 32 | 2 | 2 |
| Guangzhou 87 | 16 | 2 | 0 |
| Guangzhou 94 | 16 | 0 | 2 |
| Guangzhou 98 | 16 | 0 | 0 |
| Guangzhou 99 | 16 | 0 | 2 |
| Guangzhou 103 | 16 | 0 | 2 |
| Guangzhou 114 | 16 | 0 | 2 |
| Guangzhou 117 | 16 | 2 | 0 |
| Guangzhou 119 | 16 | 0 | 0 |
| Guangzhou 125 | 16 | 0 | 0 |
| Guangzhou 127 | 16 | 0 | 0 |
| Guangzhou 128 | 16 | 0 | 0 |
| Guangzhou 146 | 16 | 0 | 2 |
| Guangzhou 149 | 16 | 0 | 2 |
| Guangzhou 157 | 16 | 0 | 0 |
| Guangzhou 159 | 16 | 0 | 0 |
| Guangzhou 160 | 16 | 0 | 0 |
| Guangzhou 168 | 16 | 0 | 2 |
| Guangzhou 182 | 32 | 0 | 2 |
| Guangzhou 184 | 32 | 8 | 0 |
| Guangzhou 189 | 16 | 0 | 0 |
| Guangzhou 192 | 16 | 0 | 0 |
| Guangzhou 195 | 16 | 0 | 2 |
| Guangzhou 197 | 64 | 2 | 0 |
| Guangzhou 199 | 32 | 0 | 0 |
| Guangzhou 200 | 32 | 0 | 2 |
| Guangzhou 203 | 64 | 2 | 0 |
| Guangzhou 206 | 32 | 0 | 2 |
| Guangzhou 209 | 16 | 0 | 2 |
| Guangzhou 220 | 32 | 0 | 0 |
| Guangzhou 224 | 16 | 0 | 0 |
| Guangzhou 248 | 32 | 0 | 2 |
| Guangzhou 249 | 64 | 2 | 0 |
| Guangzhou 269 | 32 | 0 | 0 |
| Guangzhou 274 | 32 | 0 | 0 |
| Guangzhou 276 | 16 | 0 | 2 |
| Guangzhou 278 | 16 | 0 | 2 |
| Guangzhou 279 | 32 | 0 | 0 |
| Guangzhou 280 | 16 | 0 | 2 |
| Guangzhou 289 | 16 | 0 | 2 |
| Guangzhou 291 | 16 | 0 | 4 |
| Guangzhou 292 | 64 | 16 | 2 |
| Guangzhou 293 | 32 | 0 | 0 |
| Guangzhou 298 | 32 | 2 | 2 |
| Guangzhou 300 | 64 | 4 | 0 |
| Guangzhou 301 | 64 | 4 | 2 |
| Guangzhou 304 | 16 | 0 | 2 |
| Guangzhou 306 | 32 | 0 | 0 |
| Guangzhou 308 | 32 | 0 | 0 |
| Guangzhou 310 | 16 | 0 | 2 |
| Guangzhou 315 | 16 | 0 | 2 |
| Guangzhou 317 | 16 | 0 | 2 |
| Guangzhou 318 | 32 | 0 | 2 |
| Guangzhou 330 | 7 | 0 | 0 |
| Guangzhou 333 | 16 | 0 | 4 |
| Guangzhou 336 | 16 | 0 | 2 |
| Guangzhou 338 | 16 | 0 | 2 |
| Guangzhou 339 | 16 | 2 | 0 |
| Guangzhou 348 | 16 | 0 | 2 |
| Guangzhou 376 | 32 | 2 | 4 |
| Guangzhou 377 | 16 | 2 | 2 |
| Guangzhou 382 | 16 | 0 | 2 |
| Guangzhou 384 | 16 | 2 | 2 |
| Guangzhou 390 | 16 | 0 | 2 |
| Guangzhou 391 | 64 | 16 | 0 |
| Guangzhou 93 | 16 | 32 | 2 |
| Guangzhou 174 | 2 | 2 | 16 |
| Guangzhou 256 | 2 | 2 | 16 |
| Qingdao 4 | 16 | 0 | 4 |
| Qingdao 6 | 32 | 16 | 2 |
| Qingdao 17 | 16 | 2 | 2 |
| Qingdao 26 | 32 | 0 | 2 |
| Qingdao 29 | 16 | 2 | 2 |
| Qingdao 46 | 16 | 0 | 2 |
| Qingdao 125 | 16 | 2 | 2 |
| Qingdao 178 | 16 | 2 | 2 |
| Qingdao 35 | 2 | 2 | 16 |
| Qingdao 107 | 2 | 2 | 16 |
